# Considerations for regulation and evaluation of digital mental health technologies

**DOI:** 10.1177/20552076241293313

**Published:** 2024-11-04

**Authors:** Gareth Hopkin, Richard Branson, Paul Campbell, Holly Coole, Sophie Cooper, Francesca Edelmann, Mark Salmon

**Affiliations:** 1 Science Evidence and Analytics Directorate, National Institute for Health and Care Excellence (NICE), Manchester, England, UK; 2 Software Team, Healthcare Quality and Access Group, Medicines and Healthcare products Regulatory Agency, London, England, UK

**Keywords:** regulation, health technology assessment, evaluation, software as a medical device, SaMD

## Abstract

Digital mental health technologies (DMHTs) are becoming well established within mental health services and through direct-to-consumer models. Due to their scalable nature, DMHTs may support services to bridge the gap between demand and the available workforce, particularly where existing pathways have long delays or restricted capacity. Challenges and risks associated with DMHTs also need consideration. Regulatory and health technology assessment (HTA) agencies play a key role in ensuring the potential of DMHTs is achieved and their risks are mitigated. However, the nature of DMHTs and advances in digital technology present challenges for regulation and evaluation.

In this paper, we describe eight key considerations across the regulatory and HTA pathway for DMHTs. These relate to 1) intended purpose, 2) qualification and classification, 3) risk management, 4) clinical evidence, 5) resource requirements and economic evidence, 6) post-market surveillance and life cycle assessment, 7) replicability and equity, and 8) wider responsibilities. Ensuring clarity within these considerations and addressing outstanding uncertainties is necessary to ensure that the benefits of DMHTs are unlocked, while also ensuring that people have access to high quality and safe tools.

## Introduction

Digital mental health technologies (DMHTs) are playing an increasingly important role within mental health services. These tools can also be accessed directly by users to support their mental health.^1,2^ DMHTs are available for a range of mental health conditions and can play a role in triaging and diagnosing, providing treatment, and helping people to track and manage their mental health over time.^3^ DMHTs are also becoming increasingly complex with new generative artificial intelligence (AI) approaches coming to market.^4^

Health systems globally are looking to these opportunities to support improvements within mental health care and manage increasing system pressures.^5^ DMHTs may support services to expand access, particularly where existing pathways have long delays or restricted workforce capacity. New approaches to care may also unlock potential for earlier identification of mental health problems and provide additional tools to support existing care arrangements. The flexibility of DMHTs may also have other benefits in improving choice and supporting people who have previously struggled to access services.

Alongside these ambitions, the challenges and risks associated with DMHTs also need consideration.^6^ In a rapidly developing market, it can be unclear who technologies are aimed at, how they function, and whether claims of safety and effectiveness are supported by evidence. There are also concerns about high numbers of users discontinuing use of DMHTs early and the impact this may have on future help-seeking behaviour. Further, some randomised evidence suggests that outcomes may be worse for people using DMHTs.^7^

Regulatory and health technology assessment (HTA) agencies play a key role in ensuring the potential of DMHTs is achieved and their risks are mitigated. Regulatory agencies are responsible for ensuring the safety of medical devices and providing requirements that developers must meet for their products to be placed on the market (e.g., the Medicines and Healthcare products Regulatory Agency (MHRA) in the UK and the Food and Drug Administration (FDA) in the United States). This includes DMHTs that are considered Software as a Medical Device (SaMD). For DMHTs that are designed to be used within health services, HTA agencies or other decision-makers evaluate the effectiveness and cost-effectiveness of technologies compared with established care to make recommendations on their use (e.g., the National Institute for Health and Care Excellence (NICE) in England). However, DMHTs can present challenges for these processes due to their distinct nature, and there have been calls to clarify regulatory and HTA requirements for DMHTs.^8,9^

The aim of this article is to present key considerations across the pathway of regulation and evaluation, in order to highlight challenges relating to DMHTs, to identify where more discussion is needed, and to begin providing clarity in these areas. These considerations have been developed based on the expertise of the research team and colleagues within their respective organisations and a series of activities within a wider research project supported by Wellcome. Further, the considerations have been informed and refined through discussions with an expert working group that supports the project. Across these activities, the perspectives of stakeholders from regulatory and HTA agencies, health and care services, academia, and industry, and people with lived experiences. More details on each of the completed activities are available in Supplementary Material.

## Key considerations across the regulatory and evaluation pathway

We have identified eight key considerations across the regulatory and HTA pathway for DMHTs. These relate to 1) intended purpose, 2) qualification and classification, 3) risk management, 4) clinical evidence, 5) resource requirements and economic evidence, 6) post-market surveillance and life cycle assessment, 7) replicability and equity, and 8) wider responsibilities.

### Intended purpose

A clear account of a DMHT's intended purpose is essential for successfully navigating the regulatory requirements for medical devices and helping stakeholders understand their appropriate use and supporting evidence base. Within the UK, MHRA guidance outlines the need for developers to provide sufficient information on the 1) function, including the clinical objective(s) and how the device assists with achieving this, 2) population (people who benefit from the device), 3) who will use the device and what expertise and training is needed, 4) the operating environment in which the device is used.^10^ In our experience, many DMHTs have insufficient detail in their intended purpose statements. Through engagement with stakeholders, we identified similar experiences of DMHTs with vague or overly broad intended purposes.

There could be several reasons for this lack of detail in intended purposes. First, lack of understanding of what is required and what is useful for those choosing an appropriate DMHT with evidence. Second, mental health is complex and there may be specific challenges with defining user populations. This may be the case for DMHTs which use transdiagnostic approaches or try to avoid using medical diagnoses due to concerns about mental health stigma or over-medicalising symptoms relating to wellbeing. Last, DMHTs rapidly change and intended purposes may not be updated adequately with each new version.

Stakeholders suggested that developers are able to provide clearer accounts of their intended purpose or a more focused use-case when prompted by adopters within health services, based on where the evidence of effectiveness is strongest or where the developer is able to provide the strongest case that they provide value for money. However, for direct-to-consumer DMHTs that do not have contact with adopters within health services, intended purposes may remain overly broad due to the aim of targeting the largest number of people possible.

### Qualification and classification

A key step to bringing a product to market is determining whether a DMHT qualifies as SaMD and its appropriate classification under medical device regulations within a specific jurisdiction.^11^ Within mental health, decisions on qualification as SaMD and classification can be difficult. There are opportunities for regulatory agencies to provide clarity in this area through additional guidance and examples specific to DMHTs.

Mental health conditions are spectrum-based, meaning that symptoms can occur on a continuum including differing forms and severity. People may move between stages of this continuum more frequently than in other conditions. Further, problems closely associated with mental health conditions (e.g., poor sleep) can also occur in people without these specific conditions. This presents difficulties in assessing whether a DMHT has a medical purpose and could qualify as SaMD, or whether it is aimed at improving general wellbeing.

There is developing consensus within the DMHT community that qualification is appropriately based on the nature of symptoms being targeted (e.g., by using appropriate thresholds on clinical measures) and/or by the context that DMHTs are used in (e.g., use within defined clinical pathways). Further, qualification may depend on whether a DMHT has sufficient functionality. Some DMHTs have a medical purpose but if they have limited functionality, they may well not qualify as SaMD.

There are also challenges with determining classification. The International Medical Devices Regulatory Forum (IMDRF) principles for classification recommend devices are categorised based on the severity of the healthcare state or condition (i.e., critical, serious, non-serious) and the significance of the information (i.e., diagnosing or treating, driving clinical management, or informing clinical management).^12^ However, there is uncertainty around how severity and clinical risk should be defined within mental health conditions because symptoms can rapidly fluctuate, and the risk of negative outcomes, including self-harm and suicide, vary a lot between individuals. Further, there are challenges in defining when DMHTs play a role in treating compared with driving or informing clinical management.

### Risk management

DMHTs have the potential to improve mental health care and could either be used alongside existing services or could provide an alternative option. Developers need to demonstrate that they are able to manage clinical risk in a way that is proportionate to the intended purpose of their technology and is aligned to usual arrangements for managing clinical risk. This is likely to be particularly important for DMHTs which are used without ongoing professional support or are used outside of clinical pathways where professionals would have at least some oversight. To meet medical device regulations in the UK and in other jurisdictions, all medical devices require a risk management system that identifies and reduces risks as far as possible.

Risks may be elevated when users select products that are not designed for their circumstances and technologies do not have risk management systems that allow this to be identified and responded to appropriately. For example, there is a substantial risk of harm if a person experiencing a mental health crisis accesses a technology aimed at people with mild-to-moderate symptoms and the technology is not able to identify and appropriately signpost the user to appropriate services (e.g., an emergency telephone number or an emergency department).

Another important issue is the impact of negative experiences with DMHTs on future help-seeking behaviour. DMHTs can have high rates of drop out and low engagement by users.^13^ There are concerns that people who access digital therapies and disengage may generalise these experiences to other types of therapy or support from mental health services. This may influence help-seeking behaviour and interaction with services in the future.

### Clinical evidence

All DMHTs that qualify as SaMD need clinical evidence to show that benefits outweigh risks. This can be demonstrated through a literature review of equivalent devices and/or clinical investigations notified to the MHRA.^14^ DMHTs that are intended for use within health services may need further assessment to assess their clinical effectiveness compared with existing care and their impact on outcomes that matter to patients. In England, NICE would fulfill this role and make recommendations for use in the NHS.^15^

It has been suggested that requirements for developing clinical evidence could be a barrier to innovation and adoption due to the upfront costs and time required to generate evidence. It is therefore important to balance requirements for demonstrating that DMHTs are safe and effective, with what is achievable within sustainable business models so that DMHTs can reach patients and provide benefits. Part of ensuring this balance is likely to be clarity around expectations for developers from regulators, HTA agencies, and adopters within health services so that evidence can be developed efficiently and DMHTs can be adopted in a timely manner.

There are published frameworks to support consideration of what evidence is needed to support DMHTs. Engagement with stakeholders has suggested that the NICE Evidence Standards Framework for Digital Health Technologies (ESF) represent an acceptable balance between providing evidence and encouraging innovation.^16,17^ The NICE ESF outlines that study designs should be appropriate to support claims of benefit made by developers and should be proportional to the role a digital health technology plays. This means that it recommends that digital health technologies that are aimed at treating conditions should be supported by high-quality randomised or quasi-experimental designs. Familiarity with these frameworks and other sources of best-practice on conducting and reporting research of evidence to support assessment of effectiveness and rates of adverse events, like consensus-based preferred reporting items, will ensure that DMHTs are supported by robust and transparent evidence.

A central consideration with developing clinical evidence for DMHTs is understanding whether there is core “active ingredient” that requires evidence (e.g., therapeutic content) or whether a DMHT as a whole, including the interface and engagement features, requires evidence. Similarly, there are challenges in determining to what extent evidence from well-defined evidence-based models of therapy can be generalised to digital approaches based on these models. This has implications for evidence that is needed to support adherence to regulatory requirements and decisions on use within health services. It is also likely to have an impact on when DMHTs are assessed to have changed substantially and may need to provide updated evidence.

### Resource requirements and economic evidence

DMHTs that are designed to be used within clinical pathways in health services may need to provide evidence on their cost-effectiveness and value for money compared with existing care.^18^ In England, NICE may assess the cost-effectiveness of DMHTs, and other countries have HTA agencies that follow a similar approach. In order to support these evaluations, evidence is needed on the resources required to implement DMHTs and their influence on resource use across a pathway.

A key aspect of DMHTs’ potential is that they can widen access to support and reduce input from mental health or other professionals. This may allow services to be delivered in a more efficient way and could allow resources to be re-allocated to other areas of need. However, there will be costs associated with purchasing and maintaining DMHTs and many DMHTs require input from professionals, either to support guided self-help approaches or as an adjunct or additional tool within existing arrangements (e.g., using virtual reality during in-person sessions). At present, there appears to be a lack of consideration of these issues and uncertainty around licensing costs and additional resources needed to manage DMHTs over time and train professionals in their use. This may be particularly problematic in the context of services with high levels of staff turnover.

These upfront and ongoing costs could be outweighed if DMHTs can help release resources or there are downstream benefits and cost-savings associated with their use. Within primary care, DMHTs may play a role in reducing the need for face-to-face CBT after a period on a waiting list or may reduce staff time and allow professionals to see more patients. More substantial cost-savings may be present if DMHTs could help reduce unplanned contacts with services or admissions to hospital. However, it is uncertain to what extent these downstream impacts can be achieved and more economic evidence is needed to ensure that adoption of DMHTs does not place further strain on mental health services.

### Post-market surveillance and life-cycle assessment

DMHTs that qualify as SaMD need to be monitored after reaching the market to ensure that they remain safe and effective over time. In the UK, developers of SaMD are required to report events that have led or might have led to serious deterioration in health to the MHRA. They are also required to report any significant changes to their product to the MHRA and relevant conformity assessment bodies.^19^ The MHRA provides examples of indirect harms relating to DMHTs within their guidance. However, consistent methods of identifying and categorising adverse incidents are not being implemented.

Engagement with stakeholders suggests that there are challenges in identifying adverse events in the absence of clear requirements and agreed definitions. There is a lack of awareness of existing systems for reporting harms for DMHTs, such as yellow card reporting. In addition, there is also a need for new approaches to proactively obtain data on adverse incidents and to assess the link between DMHT use and events in multifactorial situations. This could include capturing information from a wide range of sources that includes both traditional sources (e.g., health professional reporting, yellow card) and non-traditional approaches (e.g., product reviews).

DMHTs also present new challenges due to their tendency to change quickly and significantly over time. HTA agencies typically publish a recommendation which is then reviewed periodically or when significant new information becomes available. However, DMHTs may undergo substantial changes both in the period after initial evidence has been generated and after a HTA recommendation has been made. Agencies will need to consider how they manage these issues and whether adaptations to current methods and processes are needed. For example, whether version numbers of DMHTs at the time of evidence generation and at the time of recommendations should be more prominent.

### Replicability and equity

At surface level, some DMHTs appear to be highly replicable and often share common features and similar content. There are also fewer barriers to developing software compared with other types of medical devices. For example, software products do not need to be physically manufactured and distributed. However, there are important differences such as slight variations in treatment approaches and content, the user interface, how the technologies are developed, how they manage risk, and how they are monitored over time and this has a bearing on whether DMHTs are truly comparable.

There is a lack of understanding around what features within DMHTs have clinical impact. Due to this, it is difficult to make a case that individual DMHTs which appear similar to other DMHTs are equivalent and will have a similar level of safety and effectiveness. A better understanding of the key features that drive effectiveness and safety would allow assessment of whether certain DMHTs are considered regulatory equivalent, and therefore whether bespoke evidence is required.

There may be advantages to the availability of multiple similar DMHTs. At present, DMHTs are often developed by small and medium enterprises. These companies may find it challenging to scale to the size needed to provide services for the whole population of the UK. Therefore, a number of similar products with a similar purpose may be needed to manage demand. However, as outlined above, these DMHTs are likely to need to demonstrate that an equal service is being provided.

The use of digital technologies within mental health services also has significant implications for health equity. DMHTs may improve health equity by providing more flexible approaches to care that can be accessed when it is convenient for users. However, there may also be groups of people who find DMHTs more difficult to use due to lower digital literacy and confidence and who may not be able to access them due to barriers relating to disability or socioeconomic factors. It is important that both dimensions of the impact of DMHTs on health equity are considered during development and adoption and this has been recognised as a priority across the field of digital health.^20^

### Wider responsibilities

The focus of these considerations is based on medical device regulations and health technology assessment for use within health services. However, it is important to recognise a whole system approach is needed to ensure safe and effective use of DMHTs and developers may need to be compliant with a wider range of regulations.

Other statutory regulators will have a role in ensuring that data privacy is handled appropriately, that DMHT claims and advertising across all forms of media are consistent with advertising standards, that medical practitioners and other health professionals interactions are appropriate, and that developers providing a service within DMHTs fulfil their obligations. There may also be a role for non-statutory professional bodies which represent the interests of their members and can provide professional accreditation.

There are also other processes for evaluating the evidence supporting DMHTs and their appropriateness. These may be embedded within health services as part of commissioning and procurement or this may be provided by external organisations that provide review of DMHTs. Further, there are attempts across settings to assess how reimbursement mechanisms can support adoption and to develop payment models to share risk between developers and adopters. Alignment across this full pathway is needed to ensure that innovations can be adopted and provide benefits for people experiencing poor mental health.

There are also other parties that have responsibilities in this area. DMHTs are often accessed through app stores which have historically had limited legal responsibilities to check whether apps are compliant with medical device regulations. However, laws relating to digital health are in development in several jurisdictions and this may facilitate use of high quality DMHTs.

## Discussion

The use of DMHTs presents an opportunity to improve quality and access to mental health care. However, for this potential to be unlocked stakeholders from across the system must work together to manage risk and ensure that DMHTs are adopted in a responsible way. Within this paper, we describe a series of considerations across the pathway of regulation and HTA from the development of DMHTs to monitoring their performance and safety across their lifecycle.

These issues should be considered by developers to ensure that it is clear who DMHTs are aimed at, that they have appropriate regulatory approval, and can provide evidence to support their effectiveness and their ability to mitigate risks. They should also be considered by people working within health services during implementation. Working together, these stakeholders should then be in a strong position to ensure that DMHTs are used in populations where they are appropriate, risks can be managed and that people who require other forms of support are not exposed to potential harms from misuse. Within the UK, the MHRA and NICE are collaborating on providing more certainty on how regulatory requirements and methods and processes for evaluation apply to DMHTs.^21^ There are also other initiatives underway in both the UK and internationally to explore these issues.^9,22^ For some of the considerations outlined within this paper, there are opportunities for agencies to provide additional guidance to clarify how DMHTs can comply with regulation. For example, guidance on how to assess whether DMHTs qualify as SaMD. For others, there will need to be more comprehensive initiatives to build consensus across developers, adopters, and people with lived experience to ensure that robust and proportionate approaches can balance timely access to innovative technologies and management of risk.

It is important to note that there may be limitations to the generalisability of the considerations presented in this article. The considerations have been identified from the perspective of the MHRA, which has a UK wide remit, and NICE, which is England's HTA agency. In line with this, supporting references are focused on guidance from these agencies. The considerations presented in this paper are likely to be applicable across settings and other agencies have equivalent guidance for their jurisdictions.

However, there may be additional considerations or nuances in the considerations presented here that are dependant on regulatory and evaluation pathways in other countries. For example, in some jurisdictions, regulatory agencies may exercise enforcement discretion and take a more light touch approach to DMHTs which qualify as SaMD but are low risk to users.^23^ Similarly, different jurisdictions have different processes for evaluating effectiveness of DMHTs and different levels of evidence or additional considerations may be required to be recommended for adoption or reimbursement within a specific health system.^24^

The considerations presented here are relevant to a wide range of digital mental health technologies and apply to technologies at the cutting edge of innovation including artificial intelligence. The considerations are also broad enough that they are likely to cover issues that arise from new technologies and their use within mental health. A further limitation is that it is not possible to fully predict the future trajectory of digital mental health and new technologies may present new challenges that cannot be anticipated here.

## Conclusions

DMHTs are becoming well established within mental health services and through direct-to-consumer models. The considerations presented here should be considered by developers, adopters, and other stakeholders across the system to ensure that the potential of these DMHTs can be harnessed and that risks associated with their use can be managed. Over the coming period, the considerations presented here will become better defined and new guidance will provide additional clarity and all stakeholders should engage with these efforts.

## Supplemental Material

sj-docx-1-dhj-10.1177_20552076241293313 - Supplemental material for Considerations for regulation and evaluation of digital mental health technologies Supplemental material, sj-docx-1-dhj-10.1177_20552076241293313 for Considerations for regulation and evaluation of digital mental health technologies by Gareth Hopkin, Richard Branson, Paul Campbell, Holly Coole, Sophie Cooper, Francesca Edelmann and Mark Salmon in DIGITAL HEALTH
