# Supplementary material for: Considerations for regulation and evaluation of digital mental health technologies
Source: Digit Health. 2024 Nov 4;10:20552076241293313. doi: 10.1177/20552076241293313 (PMC11536580; doi:10.1177/20552076241293313)
Supplement: sj-docx-1-dhj-10.1177_20552076241293313 - Supplemental material for Considerations for regulation and evaluation of digital mental health technologies [file sj-docx-1-dhj-10.1177_20552076241293313.docx]

Appendices

# Appendix 1 - Activities to support development of considerations

**Background**

We completed a series of reviews and activities to identify key considerations across the regulatory and HTA pathway. This has included a regulatory review of data on DMHTs at touchpoints with the MHRA, a review of assessments by NICE, a review of DMHTs within apps stores and app store policies, and roundtables and other engagements with international regulators, industry, professionals and academic involved in delivering care, and organisations supporting health innovation.

**Information sources**

*Regulatory review*

We reviewed DMHT data at touchpoints with the MHRA across the regulatory pathway. This included information: 1) submitted to the MHRA as part of clinical investigations of non-UKCA / CE marked medical devices, 2) from medical device registrations to place products on the UK market, 3) adverse incidents, and 4) regulatory enquiries,

*Health technology assessment review*

We reviewed all DMHTs that have been evaluated through NICE early value assessment (EVA) and medical technologies guidance (MTG) programmes and were published at the time of review. Within NICE EVA, this included 23 DMHTs that were within scope of five appraisals on self-guided and guided therapies for children and adults with anxiety and depression, virtual reality for agoraphobia, and management of psychosis. Within NICE MTG, this included one DMHT reviewed as part of a single appraisal.

*Roundtables*

We convened a series of roundtables with subject matter experts from a range of clinical and academic disciplines to explore a series of questions relating to DMHTs. These roundtables included professionals from international regulators, working within frontline health services and academia, professionals working within industry for developers or other representative bodies, and professionals supporting innovation and adoption across the health system.

*Export working group discussions*

Based on the reviews described above, we developed a series of initial considerations and presented these to the project expert working group across several meetings. We asked members to reflect on our initial findings and specific considerations within small breakout rooms. These reflections were then fed back to the larger group and initial findings from the reviews were updated according to the perspectives and experiences of experts within the working group.

The working group is a standing group of experts that advise the project and comprises representatives from the MHRA and NICE (including members of the project team and other experts from these organisation), representatives from NHS England, a variety of healthcare professionals with experience of providing mental health care (e.g. clinical psychology, nursing, psychiatry, general practice), academics, members of health innovation networks, and lived experience advisers.
